# Emergency Department Visits by Patients with Substance Use Disorder in the United States

**DOI:** 10.5811/westjem.2021.3.50839

**Published:** 2021-08-19

**Authors:** Xingyu Zhang, Ningyuan Wang, Fengsu Hou, Yaseen Ali, Aaron Dora-Laskey, Chin Hwa Dahlem, Sean Esteban McCabe

**Affiliations:** * University of Michigan School of Nursing, Department of Systems, Populations, and Leadership, Ann Arbor, Michigan, United States of America; † University of Michigan, College of Literature, Science, and the Arts; Ann Arbor, Michigan, United States of America; ‡ Shenzhen Kangning Hospital, Department of Public Health, Shenzhen, Guangdong Province, China; § Michigan State University College of Human Medicine, Department of Emergency Medicine, East Lansing, Michigan, United States of America; ¶ University of Michigan School of Nursing, Department of Health Behavior and Biological Sciences, Ann Arbor, Michigan, United States of America; || University of Michigan, Institute for Social Research, Ann Arbor, Michigan; # University of Pittsburgh Medical Center, Thomas E. Starzl Transplantation Institute, Pittsburgh, Pennsylvania, United States of America

## Abstract

**Introduction:**

We aimed to characterize emergency department (ED) utilization and clinical characteristics of patients with substance use disorder (SUD) seeking emergency care for all reasons.

**Methods:**

Using 2016–2017 ED data from the National Hospital Ambulatory Medical Care Survey, we investigated demographics, ED resource utilization, and clinical characteristics of patients with SUD vs those without SUD.

**Results:**

Of all adult ED visits (N = 27,609) in the US in 2016–2017, 11.1% of patients had SUD. Among ED patients with SUD, they were mostly non-Hispanic White (62.5%) and were more likely to be male (adjusted odds ratio [aOR] 1.80 confidence interval [CI], 1.66–1.95). Emergency department patients with SUD were also more likely to return to the ED within 72 hours (aOR 1.32, CI, 1.09–1.61) and more likely to be admitted to the hospital (aOR 1.28, CI, 1.14–1.43) and intensive care unit (aOR 1.40, CI, 1.05–1.85).

**Conclusion:**

Patients with SUD have specific demographic, socioeconomic, and clinical characteristics associated with their ED visits. These findings highlight the importance of recognizing co-existing SUD as risk factors for increasing morbidity in acutely ill and injured patients, and the potential role of the ED as a site for interventions aimed at reducing harm from SUD.

## INTRODUCTION

Studies have shown people with substance use disorders (SUD) are more likely to experience trauma, report lower quality of life, and be diagnosed with mental illness, cancer, and heart disease.^1^–^3^ According to the 2017 National Survey on Drug Use and Health,^4^ almost 74% of adults with a SUD had an alcohol use disorder and approximately 38% of adults with a SUD had an illicit drug use disorder. Substance use, misuse, and SUD cost American society more than $740 billion annually in lost workplace productivity, healthcare expenses, and crime-related costs.^5^–^10^

Substance-related injuries, soft tissue infections, and overdoses often result in admissions to the emergency department (ED), and therefore provide a window of opportunity to identify and connect people with SUD for treatment and referral services.^11^,^12^ Previous studies found that socioeconomic status influenced a person’s substance use.^13^–^16^ However, there is limited research using nationally representative samples to examine the association between patients with SUD and the characteristics of their ED visits.^17^ Through better understanding the medical care needs of people with SUDs, EDs can be the bridge to connect patients to evidence-based interventions to the community upon discharge.^18^

To better understand the relationship between SUDs and ED visits, we conducted a secondary analysis of a large nationally representative dataset. In particular, the current study aimed to do the following: 1) estimate ED use by patients with SUD; 2) characterize the clinical presentation of ED patients with SUD; and 3) examine factors associated with clinical outcome and resource utilization for ED patients with SUD. The goal of the study was to provide information that could potentially improve quality of ED care delivered to patients with SUD.

## METHODS

### Study Population

We performed a cross-sectional study on the adult patients (age≥ 18 years) (N = 27,609) in the National Hospital Ambulatory Medical Care Survey-Emergency Department subfile (NHAMCS-ED) from 2016–2017.^19^ The NHAMCS-ED is a nationally representative, multistage, stratified probability sample of ED visits in the United States, administered by the National Center for Health Statistics, a branch of the US Centers for Disease Control and Prevention.^20^ The 2016–2017 NHAMCS-ED data were collected from about 600 hospital-based EDs across all 50 states. The NHAMCS-ED survey uses a standardized template to collect detailed information from approximately 100 patients per hospital-based ED annually.

This study was determined to be exempt by the institutional review board since we used publicly available data.

### Study Design and Variables

The primary outcome for this study was the percentage of ED patients diagnosed with SUD. We identified SUD visits to US EDs by adults (age≥ 18 years) with a chief diagnosis or mental health condition relating to alcohol and/or other drug use disorder. The NHAMCS-ED collects up to three main diagnosis codes for ED visits and two health history codes relating to alcohol or other drug use disorder, using the *International Classification of Diseases, Tenth Revision Clinical Modification* (ICD-10-CM) codes.

A patient was classified with SUD if an alcohol or other drug use disorder was identified using two approaches during the ED visit. First, an alcohol use disorder (AUD) was considered to be present when the box on the patient record form for the question “Does the patient have alcohol misuse, abuse, or dependence?” was checked from the patient’s electronic health record. Similarly, drug use disorder (DUD) was present when the box on the patient record form for the question “Does the patient have substance abuse or dependence?” was checked by the physician.

**Population Health Research Capsule**

What do we already know about this issue?

*Substance-related health issues often result in admissions to the emergency department (ED), however, the population’s characteristics of the ED visits related to substance use disorder (SUD) haven’t been systemically studied*.

What was the research question?

*What are the factors that associated emergency department (ED) use for ED patients with substance use disorders?*

What was the major finding of the study?

*11.1% of ED patients had substance use disorder, and were more commonly male, non-Hispanic White, subject to repeat ED visits and more likely to be admitted and sent to the intesive care unit*.

How does this improve population health?

*These findings highlight the potential role of the emergency department as a site for interventions aimed at reducing harm from SUD*.

Second, we classified patients as having SUD if one of the following ICD-10-CM codes were included in the three providers’ diagnosis codes listed on the patient record form: *F10, F11, F12, F13, F14, F15, F16, F17, F18, F19*, and corresponding pediatric codes.^21^ The above codes for SUD include codes for alcohol-related disorders, opioid-related disorders, cannabis-related disorders, sedative-, hypnotic-, or anxiolytic-related disorders, cocaine-related disorders, other stimulant-related disorders, hallucinogen-related disorders, nicotine dependence, inhalant-related disorders, and other psychoactive substance-related disorders. In this study, we classified four SUD statuses: alcohol use disorder (AUD only); other drug use disorder (DUD only, including nicotine dependence); alcohol or drug use disorder (SUD); and no alcohol or drug use disorder.

Secondary outcomes included the Emergency Severity Index (ESI) score (a five-level ED triage algorithm assigning patients a score from 1 [most urgent] to 5 [least urgent] on the basis of acuity and resource needs); hospital admission; intensive care unit (ICU) admission; blood tests; imaging (including radiograph, computed tomography, ultrasound, and magnetic resonance imaging (MRI); procedures (bilevel positive airway pressure/continuous positive airway pressure; bladder catheter; cast, splint, wrap; central line other; intravenous (IV) fluids; cardiopulmonary resuscitation; endotracheal intubation; incision and drainage; IV fluids; lumbar puncture; nebulizer therapy; pelvic exam; skin adhesives; suturing/staples; other); patient’s waiting time; whether the patient left before triage or treatment; and whether the patient died in the ED/hospital.

Covariates included demographic characteristics (age, gender, race/ethnicity, region); socioeconomic status indicators, including residence (private home, nursing home, homeless, other) and insurance (private insurance, Medicare, Medicaid/Children’s Health Insurance Program, uninsured, other); day and mode of arrival; triage vital signs (temperature, pain scale, blood pressure, etc); whether this visit related to an injury/trauma overdose/poisoning/adverse effect of medical/surgical treatment; and reason for the ED visit. To assign a primary reason for each ED visit, we synthesized 10 system-based symptom clusters from the nine symptom modules used in the NHAMCS (p. 23 in 2016 documentation).^22^ Note that, as per the NHAMCS modules, our “Reason for ED Visit – Psychiatric” cluster excluded the following: alcoholism; adverse effects of alcohol; drug (prescription and illicit) addiction/dependence; drug intoxication; intentional drug overdose; and unintentional overdose.

### Statistical Analyses

We described and compared population characteristics between ED patients with SUD vs those without SUD using Pearson’s chi-squared test for survey samples and Rao-Scott chi-squared test for weighted samples. After adjusting for confounding factors, we used logistic regression to test associations between SUD and the covariates. We also used logistic regression to investigate associations between SUDs and secondary outcomes, testing for mediation by covariates. The NHAMCS-ED dataset used in this analysis relies on a sequential hot-deck method to impute three-digit ICD-10-CM codes for items such as age, gender, primary diagnosis, ED volume, and geographic region. Other variables were imputed with the median of the corresponding variables prior to generating the logistic regression models. We used SAS version 9.4 (SAS Institute Inc., Cary, NC) for our analysis, setting α = 0.05 as the statistical significance threshold. All odds ratios were calculated with 95% confidence intervals (CI).

## RESULTS

The characteristics of ED visits made by SUD and non-SUD patients are shown in Table 1, Table 2, and Supplement Table 1. In 2016–2017, there were 27,609 adult ED visits in the US, and 3282 (11.1%) involved SUD. Among all ED visits that involved SUD, 18.7% involved AUD only, and 61.5% involved DUD only. The proportion of ED visits by patients with SUD varied by US geographic areas: Northeast, 15.4%; Midwest, 28.6%; South, 30.6%; and West, 25.3% (*P* <0.01).

The gender and race/ethnicity distributions of the sample varied across diagnostic groups. Male patients were more likely to have a SUD than females. Furthermore, non-Hispanic White patients were more likely to receive a diagnosis than other races or ethnicities. Patients aged 26–39 were more heavily represented in DUD only, while ED patients aged 50–59 were more likely to be diagnosed as AUD only.

Table 3 describes the association between ED patients’ characteristics (demographic, socioeconomic, and clinical) and their SUD status using multiple logistic regression analyses. We found that male patients with SUD status were more likely to be frequent users of EDs than female patients with SUD (adjusted odds ratio [aOR] 1.79, CI, 1.66–1.94). Among ED patients, Asians were 50% less likely (aOR 0.50, CI, 0.34–0.73) than non-Hispanic Whites to be diagnosed with a SUD. Among all SUD status patients, compared to ED patients inhabiting a private residence, homeless patients were 4.04 (aOR 4.04, CI, 3.29–4.96) times more likely to be in SUD status, while people living in nursing homes were 68% (aOR 0.32, CI, 0.20–0.52) less likely to have SUD. In terms of mode of arrival, ED visits by patients with SUD were 2.29 (aOR 2.29, CI, 2.09–2.52) times more likely to arrive via emeregency medical services.

Further, ED patients with AUD were 3.36 (aOR 3.36, CI, 2.95–3.82) times more likely to arrive by ambulance, which was much higher than the ED patient with DUD. In terms of physical characteristics of ED visits, among ED patients with SUDs they were more likely to have faster heart rates compared to heart rates under 90 beats per minute (heart rate in 90–100, CI, 1.16–1.43; heart rate in 100–110, CI, 1.22–1.58; heart rate in 110–120, CI, 1.32–1.85; heart rate over 120, CI, 1.60–2.35). In addition, ED patients with SUDs were 1.32 (aOR 1.32, CI, 1.09–1.61) times more likely to have a revisit within 72 hours. Regarding reasons for ED visits classified by symptom modules, ED patients with SUD were 3.08 (aOR 3.08, CI, 2.62–3.62) times more likely to present with psychiatric symptoms than general symptoms, and their ED visits were 1.19 (aOR 1.19, CI, 1.07–1.33) more likely to be related to injury.

Table 4, Table 5, and Supplement Table 2 illustrate the association of ED patients’ characteristics (ESI score, hospital admission, ICU admission, and medical resources utilization) and their status on SUD; the association has been adjusted by demographic, socioeconomic, and clinical confounding factors. We found ED visits of patients with SUD were less likely to apply imaging diagnoses. For example, radiograph was 11% (aOR 0.89, CI, 0.82–0.97) less likely to be used for ED visits of patients with SUD. Other imaging diagnosis examinations, such as ultrasound and MRI, showed similar trends for patients with SUD. More details are shown in Table 5. Additionally, ED patients with SUD tended to have a higher hospitalization rate; they were 1.28 (aOR 1.28, CI, 1.14–1.43) and 1.40 (aOR 1.40, CI, 1.05–1.85) times more likely to be hospitalized and admitted to the ICU, respectively. In addition, ED visits by patients with SUD were 1.31 (aOR 1.31, CI, 1.15–1.49) times more likely to have mortality in the ED compared to other ED visits.

## DISCUSSION

We present a comprehensive study describing the national characteristics of ED patients with SUD. As opposed to previous studies,^17^,^23^ we used a more recent national sample and included adult patients with both SUD medical history and with a SUD diagnosis at the current ED visits. It is estimated that out of 5.1 million drug-related ED visits, nearly one-half (49%) were due to drug misuse or abuse.^17^ Thus, the ED can be the initial entry point for people with SUD to receive and be referred for treatment and recovery support services. The study by Moulin et al showed that people with SUDs are more likely to experience homelessness, suffer from mental illness, require ambulance services, and return to the ED than people without SUDs^24^; and the results from the current study are consistent with these findings. Further, patients with SUD were more likely to be hospitalized and admitted to the ICU and to experience higher mortality than people without SUDs.

We found that male ED patients were more likely than females to be diagnosed with SUD, as were non-Hispanic Whites compared to other races/ethnicities, particularly Asians. These genders and racial/ethnic differences are consistent with demographic patterns in SUDs observed beyond the ED setting in a survey of psychiatrists treating patients with SUDs.^25^ Compared to non-SUD patients in ED visits, patients with SUD in the ED are more likely to be uninsured. It is worth noting similarities between ED patients with SUD and ED patients with cancer, whose utilization is higher across many dimensions of care.^26^,^27^ People living in nursing homes were less likely to have SUD as nursing homes are largely profit-driven enterprises and tend to not accept (or remove) patients with SUD.

It is also noticeable that ED patients with SUDs have a higher chance of revisiting the ED within 72 hours. Further examining the reasons for return ED visits among patients with SUD can facilitate the development of interventions and guidelines to improve the quality of care for people with SUDs. For instance, a study by Barata et al^28^ showed that ED-based interventions for people with AUD can reduce alcohol use and repeat ED visits. Additionally, ED-based initiation of buprenorphine for opioid use disorder with primary care follow-up was shown to increase treatment engagement and decrease self-reported, seven-day opioid use.^11^ Thus, given the substantial number of patients with SUD who frequent the ED, the ED remains a promising and understudied setting for linking people with SUD to care and treatment.

Our study advances understanding of characteristics and clinical performance of patients with SUD in the ED setting. It is an initial step toward establishing a baseline and improving this population’s care and clinical outcomes in the ED and further reducing their ED burden. The study revealed the characteristics of ED patients with SUD in a diverse, national sample. In the ED, patients with SUD have significantly higher hospital admission, ICU admission, and mortality compared to those without SUD, indicating that patients with SUD may require a better understanding and higher level of emergency care and services. These findings argue for increasing recognition of the potential of the ED as a high-leverage setting for improving treatment and screening of SUD, by identifying characteristics and trajectories of patients presenting to the ED with SUD.

## LIMITATIONS

Several limitations of this study should be noted. First, the data were unable to differentiate the drug-specific types of SUD exhibited by each patient with SUD in ED visits. Based on patient histories documented in the NHAMCS-ED data, patients with SUD were coded as AUD Only, DUD Only, and SUD. More information about the drug-specific type of SUD would allow for more characteristics of ED visits by adult patients with SUD to account for different drug patterns.^29^, ^30^ Second, study data came from hospitals in the NHAMCS-ED database and the variables available for analysis were limited. Information such as reasons and duration of SUD for those patients would have been optimal. The data provided for the analysis was only from 2016–2017, and it was limited to illustrate trends and patterns of national characteristics of ED visits among patients with SUDs over time. And, finally, the SUD cases might be under-reported due to the degree of accuracy of diagnosis in the ED.

## CONCLUSION

This study describes the clinical characteristics of ED utilization in patients with substance use on a national scale, which enhanced our understanding of the characteristics of this population. We detected gender, racial/ethnic, and economic differences between ED patients with and without substance use disorder. Patients with SUD are more likely to be admitted to the hospital and ICU and are more likely to return to the ED. The findings highlight the importance of recognizing co-existing SUD as a risk factor for increased morbidity in acutely ill and injured patients, and the potential role of the ED as a site for interventions aimed at reducing harm from SUD.

## Supplementary Information



## Figures and Tables

**Table 1:** Baseline characteristics of patients presenting to the emergency department, stratified by alcohol/drugs substance use disorder, *NHAMCS 2016–2017(unweighted sample).

	All	DUD only	AUD only	SUD (DUD or AUD)	No SUD
	27,609	2,668(9.7)	1,265(4.6)	3,282(11.9)	24,327(88.1)
Male	2,031(43.6)	1,519(56.9)**	870(68.8)**	1,926(58.7)**	10,105(41.5)
Age		**	**	**	
18–25	3,978(14.4)	402(15.1)	106(8.4)	449(13.7)	3,529(14.5)
26–39	7,598(27.5)	884(33.1)	300(23.7)	1,025(31.2)	6,573(27.0)
40–49	4,237(15.4)	505(18.9)	243(19.2)	624(19.0)	3,613(14.9)
50–59	4,338(15.7)	531(19.9)	384(30.4)	705(21.5)	3,633(14.9)
60–74	4,496(16.3)	293(11.0)	210(16.6)	408(12.4)	4,088(16.8)
≥ 75	2,962(10.7)	53(2.0)	22(1.7)	71(2.2)	2,891(11.9)
Race/ethnicity		**	*	**	
NH White	12,731(60.3)	1,226(63.2)	544(58.0)	1,494(62.5)	11,237(60.0)
H White	1,550(7.3)	102(5.3)	61(6.5)	137(5.7)	1,413(7.5)
NH Black	4,796(22.7)	450(23.2)	233(24.8)	557(23.3)	4,239(22.6)
H Black	70(0.3)	9(0.5)	4(0.4)	11(0.5)	59(0.3)
Hispanic (Other)	1,096(5.2)	81(4.2)	61(6.5)	109(4.6)	987(5.3)
Asian	548(2.6)	27(1.4)	15(1.6)	33(1.4)	515(2.8)
Other	325(1.5)	45(2.3)	20(2.1)	51(2.1)	274(1.5)
Residence type		**	**	**	
Private residence	25,607(94.7)	2,303(89.0)	1,029(84.7)	2,829(88.8)	22,778(95.5)
Nursing home	506(1.9)	10(0.4)	12(1.0)	19(0.6)	487(2.0)
Homeless	485(1.8)	209(8.1)	138(11.4)	256(8.0)	229(1.0)
Other	434(1.6)	66(2.6)	36(3.0)	83(2.6)	351(1.5)
Insurance type		**	**	**	
Private insurance	7,380(29.3)	521(21.6)	225(20.2)	635(21.6)	6,745(30.4)
Medicare	6,499(25.8)	366(15.2)	205(18.4)	494(16.8)	6,005(27.0)
Medicaid or CHIP	7,916(31.5)	1,153(47.9)	521(46.8)	1,377(46.7)	6,539(29.4)
Uninsured	2,482(9.9)	284(11.8)	123(11.0)	335(11.4)	2,147(9.7)
Other	889(3.5)	85(3.5)	40(3.6)	105(3.6)	784(3.5)
Day of ED visit		**	**	**	
Weekend	7,277(26.4)	736(27.6)	368(29.1)	907(27.6)	6,370(26.2)
Weekdays	20,332(73.6)	1,932(72.4)	897(70.9)	2,375(72.4)	17,957(73.8)
Arrive by ambulance	5074(18.9)	756(28.9)**	596(48.3)**	1,034(32.2)**	4,040(17.1)
Seen within last 72 hours	870(3.4)	121(4.9)**	73(6.2)**	157(5.2)**	713(3.2)
Pain level at presentation		**	**	**	
No pain	4,831(24.8)	517(29.3)	333(41.7)	666(30.7)	4,165(24.0)
Mild	1,868(9.6)	120(6.8)	58(7.3)	156(7.2)	1,712(9.9)
Moderate	6,019(30.8)	476(27.0)	180(22.5)	576(26.6)	5,443(31.4)
Severe	6,801(34.8)	653(37.0)	228(28.5)	770(35.5)	6,031(34.8)
Temperature at presentation	36.8(0.4)	36.7(0.4)	36.7(0.4)	36.7(0.4)	36.8(0.4)
Heart rate at presentation	85.9(17.5)	89.6(17.6)	91.9(18.7)	90.1(18.0)	85.3(17.4)
DBP at presentation	80.4(14.7)	82.0(14.7)	82.7(15.1)	82.2(14.9)	80.1(14.6)
SBP at presentation	137.4(23.6)	134.9(21.7)	134.1(22.2)	135.0(22.0)	137.7(23.8)
Census region					
Northeast	4,503(16.3)	388(14.5)	265(20.9)	507(15.4)	3,996(16.4)
Midwest	6,756(24.5)	814(30.5)	253(20.0)	940(28.6)	5,816(23.9)
South	9,720(35.2)	835(31.3)	343(27.1)	1,004(30.6)	8,716(35.8)
West	6,630(24.0)	631(23.7)	404(31.9)	831(25.3)	5,799(23.8)
Visit related to injury	8,493(30.8)	910(34.1)**	575(45.5)**	1,192(36.3)**	7,301(30.0)

* NHAMCS, National Hospital Ambulatory Medical Care Survey.

Note: the missing proportion for residency type and arriving by ambulance is less than 5%; for insurance, temperature and seen within 72h, 5% – 10 %; for race/ethnicity, 20% – 25%; for pain level, 29%; Independent test was performed on categories of drug use disorder (DUD), alcohol use disorder (AUD), and drug or alcohol use disorder (SUD). Pearson’s chi-squared test was performed on unweighted samples, and Rao-Scott corrected chi-squared test was performed on weighted samples.

* P < 0.05,

** P < 0.01,

*NH*, non-Hispanic; *H*, Hispanic.

*CHIP*, Children’s Health Insurance Program; *ED*, emergency department; *DUD*, drug use disorder; *AUD*, alcohol use disorder; *SUD*, drug or alcohol use disorder; *DBP*, diastolic blood pressure; *SBP*, systolic blood pressure.

**Table 2:** Selected reason for visit and emergency department diagnosis among patients with drugs/alcohol substance use disorder, *NHAMCS 2016–2017.

	All	DUD only	AUD only	SUD (DUD or AUD)	No SUD
General	5,305(19.2)	533(20.0)	235(18.6)	638(19.5)	4,667(19.2)
Psychiatric	1,146(4.2)	343(12.9)	231(18.3)	428(13.1)	718(3.0)
Neurologic	2,031(7.4)	167(6.3)	77(6.1)	202(6.2)	1,829(7.5)
Cardiovascular and lymphatic	591(2.1)	47(1.8)	22(1.7)	57(1.7)	534(2.2)
Eyes and/or ears	563(2.0)	25(0.9)	4(0.3)	29(0.9)	534(2.2)
Respiratory	2,732(9.9)	202(7.6)	53(4.2)	233(7.1)	2,499(10.3)
Digestive	4,360(15.8)	382(14.3)	132(10.4)	468(14.3)	3,892(16.0)
Genitourinary	1,490(5.4)	66(2.5)	16(1.3)	78(2.4)	1,412(5.8)
Dermatologic	796(2.9)	73(2.7)	15(1.2)	81(2.5)	715(2.9)
Musculoskeletal	4,073(14.8)	315(11.8)	95(7.5)	370(11.3)	3,703(15.2)
Other	4,484(16.3)	512(19.2)	384(30.4)	695(21.2)	3,789(15.6)

* NHAMCS, National Hospital Ambulatory Medical Care Survey.

*DUD*, drug use disorder; *SUD*, substance use disorder; *AUD*, alcohol use disorder.

**Table 3:** Association between alcohol/substance use disorder status in emergency department patients and their visit characteristics (*NHAMCS 2016–2017).

	SUD (AUD or DUD)		AUD only		DUD only	
			
	Crude OR (95% CI)	Adjusted OR (95% CI)	Crude OR (95% CI)	Adjusted OR (95% CI)	Crude OR (95% CI)	Adjusted OR (95% CI)
Age						
18–25	Reference [1]	Reference [1]	Reference [1]	Reference [1]	Reference [1]	Reference [1]
26–39	1.23(1.09–1.38)	1.16(1.02–1.31)	1.50(1.20–1.88)	1.42(1.12–1.80)	1.17(1.03–1.33)	1.10(0.97–1.25)
40–49	1.36(1.19–1.55)	1.28(1.12–1.47)	2.22(1.76–2.80)	2.17(1.70–2.78)	1.20(1.05–1.38)	1.12(0.96–1.29)
50–59	1.53(1.34–1.73)	1.34(1.16–1.53)	3.55(2.85–4.42)	3.22(2.55–4.08)	1.24(1.08–1.42)	1.08(0.93–1.25)
60–74	0.78(0.68–0.90)	0.73(0.62–0.85)	1.79(1.41–2.27)	1.76(1.35–2.28)	0.62(0.53–0.73)	0.59(0.50–0.71)
≥ 75	0.19(0.15–0.25)	0.17(0.13–0.22)	0.27(0.17–0.43)	0.24(0.15–0.38)	0.16(0.12–0.22)	0.15(0.11–0.21)
Male vs female	2.00(1.86–2.15)	1.79(1.66–1.94)	3.00(2.65–3.38)	2.35(2.06–2.68)	1.82(1.67–1.97)	1.65(1.51–1.80)
Race/ethnicity						
NH White	Reference [1]	Reference [1]	Reference [1]	Reference [1]	Reference [1]	Reference [1]
White	0.73(0.61–0.88)	0.64(0.53–0.78)	0.92(0.70–1.20)	0.81(0.61–1.08)	0.66(0.54–0.82)	0.59(0.48–0.74)
NH Black	0.99(0.89–1.10)	0.76(0.68–0.85)	1.14(0.98–1.34)	1.02(0.86–1.20)	0.97(0.87–1.09)	0.72(0.64–0.81)
Black	1.40(0.74–2.68)	0.98(0.48–1.96)	1.36(0.49–3.74)	0.96(0.32–2.89)	1.39(0.69–2.80)	0.94(0.44–1.97)
Hispanic	0.83(0.68–1.02)	0.64(0.52–0.80)	1.32(1.01–1.73)	0.96(0.71–1.29)	0.75(0.59–0.95)	0.60(0.47–0.77)
Asian	0.48(0.34–0.69)	0.50(0.34–0.73)	0.63(0.38–1.06)	0.52(0.30–0.91)	0.49(0.33–0.72)	0.55(0.37–0.82)
Other	1.40(1.03–1.90)	1.21(0.88–1.68)	1.47(0.93–2.33)	1.23(0.74–2.02)	1.51(1.10–2.08)	1.35(0.97–1.89)
Day of week						
Weekdays	Reference [1]	Reference [1]	Reference [1]	Reference [1]	Reference [1]	Reference [1]
Weekends	1.08 (0.99–1.17)	1.10(1.01–1.20)	1.15(1.02–1.31)	1.19(1.04–1.37)	1.07(0.98–1.17)	1.10(1.00–1.20)
Residence type						
Private residence	Reference [1]	Reference [1]	Reference [1]	Reference [1]	Reference [1]	Reference [1]
Nursing home	0.31(0.20–0.50)	0.32(0.20–0.52)	0.58(0.33–1.03)	0.42(0.23–0.77)	0.20(0.11–0.38)	0.24(0.13–0.46)
Homeless	9.00(7.50–10.80)	4.05(3.30–4.96)	9.50(7.72–11.68)	2.76(2.17–3.53)	7.66(6.37–9.22)	3.78(3.07–4.64)
Other	1.90(1.49–2.43)	1.16(0.89–1.51)	2.16(1.53–3.06)	0.94(0.64–1.37)	1.82(1.39–2.37)	1.22(0.92–1.62)
Insurance type						
Private insurance	Reference [1]	Reference [1]	Reference [1]	Reference [1]	Reference [1]	Reference [1]
Medicare	0.87(0.77–0.99)	1.40(1.24–1.58)	1.04(0.86–1.26)	1.38(1.14–1.66)	0.79(0.68–0.90)	1.32(1.16–1.51)
Medicaid or CHIP	2.24(2.02–2.47)	2.11(1.90–2.35)	2.24(1.91–2.63)	1.89(1.59–2.25)	2.24(2.01–2.50)	2.11(1.88–2.37)
Uninsured	1.66(1.44–1.91)	1.48(1.28–1.73)	1.66(1.32–2.08)	1.50(1.18–1.92)	1.70(1.46–1.98)	1.50(1.28–1.76)
Other	1.42(1.14–1.77)	1.09(0.87–1.38)	1.50(1.06–2.11)	0.95(0.66–1.37)	1.39(1.09–1.77)	1.13(0.88–1.45)
Temperature						
36°C–38°C	Reference [1]	Reference [1]	Reference [1]	Reference [1]	Reference [1]	Reference [1]
≤ 36°C	1.37(1.11–1.68)	1.24(0.99–1.55)	1.95(1.49–2.56)	1.67(1.24–2.25)	1.30(1.04–1.63)	1.21(0.95–1.54)
> 38°C	0.53(0.35–0.80)	0.50(0.32–0.77)	0.48(0.24–0.98)	0.46(0.22–0.96)	0.49(0.30–0.79)	0.48(0.30–0.79)
Heart rate						
≤ 90	Reference [1]	Reference [1]	Reference [1]	Reference [1]	Reference [1]	Reference [1]
90–100	1.42(1.29–1.57)	1.29(1.16–1.43)	1.53(1.32–1.77)	1.51(1.28–1.77)	1.39(1.25–1.55)	1.23(1.10–1.37)
100–110	1.54(1.37–1.74)	1.39(1.22–1.58)	1.70(1.42–2.03)	1.76(1.44–2.14)	1.47(1.29–1.67)	1.27(1.11–1.46)
110–120	1.76(1.51–2.06)	1.56(1.32–1.85)	2.14(1.71–2.68)	2.19(1.71–2.81)	1.57(1.32–1.88)	1.32(1.10–1.60)
> 120	2.17(1.82–2.58)	1.94(1.60–2.35)	2.68(2.10–3.41)	2.65(2.02–3.48)	1.88(1.55–2.29)	1.61(1.30–1.98)
DBP						
60–80	Reference [1]	Reference [1]	Reference [1]	Reference [1]	Reference [1]	Reference [1]
< 60	1.01(0.88–1.15)	1.06(0.92–1.23)	1.06(0.86–1.30)	1.08(0.86–1.35)	1.01(0.87–1.17)	1.08(0.93–1.26)
> 80	1.25(1.16–1.35)	1.06(0.97–1.15)	1.30(1.15–1.46)	1.04(0.91–1.19)	1.23(1.13–1.34)	1.06(0.97–1.16)
Pain level						
No pain	Reference [1]	Reference [1]	Reference [1]	Reference [1]	Reference [1]	Reference [1]
Mild	0.57(0.47–0.68)	0.72(0.59–0.87)	0.43(0.33–0.58)	0.63(0.47–0.85)	0.57(0.47–0.70)	0.71(0.57–0.89)
Moderate	0.66(0.59–0.75)	0.91(0.82–1.02)	0.42(0.35–0.50)	0.73(0.63–0.85)	0.72(0.63–0.82)	0.97(0.86–1.09)
Severe	0.80(0.72–0.89)	0.87(0.77–0.99)	0.47(0.39–0.56)	0.61(0.50–0.74)	0.89(0.79–1.00)	0.96(0.84–1.10)
72-hour revisit vs not	0.61(0.51–0.72)	1.32(1.09–1.61)	0.51(0.40–0.66)	1.46(1.10–1.93)	0.66(0.54–0.80)	1.21(0.98–1.50)
Arrival by ambulance versus not	2.30(2.12–2.49)	2.29(2.09–2.52)	4.41(3.92–4.95)	3.36(2.95–3.83)	1.87(1.71–2.05)	1.90(1.71–2.11)
Census Region						
Northeast	Reference [1]	Reference [1]	Reference [1]	Reference [1]	Reference [1]	Reference [1]
Midwest	1.27(1.14–1.43)	1.53(1.35–1.73)	0.62(0.52–0.74)	0.76(0.63–0.92)	1.45(1.28–1.65)	1.71(1.49–1.96)
South	0.91(0.81–1.02)	1.07(0.95–1.21)	0.59(0.50–0.69)	0.70(0.58–0.84)	1.00(0.88–1.13)	1.16(1.02–1.33)
West	1.13(1.00–1.27)	1.06(0.93–1.21)	1.04(0.89–1.22)	1.05(0.88–1.26)	1.12(0.98–1.27)	1.01(0.88–1.17)
Reason for visit (by symptom module)						
General	Reference [1]	Reference [1]	Reference [1]	Reference [1]	Reference [1]	Reference [1]
Psychiatric	4.36(3.77–5.04)	3.08(2.62–3.62)	5.45(4.48–6.62)	3.26(2.62–4.04)	3.83(3.28–4.47)	2.73(2.31–3.24)
Neurologic	0.81(0.68–0.96)	0.79(0.66–0.94)	0.85(0.65–1.11)	0.86(0.66–1.14)	0.80(0.67–0.96)	0.78(0.65–0.94)
Cardiovascular and lymphatic	0.78(0.59–1.04)	0.89(0.66–1.21)	0.83(0.53–1.30)	0.88(0.55–1.40)	0.77(0.57–1.06)	0.93(0.67–1.28)
Eyes and/or ears	0.40(0.27–0.58)	0.45(0.30–0.66)	0.15(0.06–0.42)	0.22(0.08–0.61)	0.42(0.28–0.63)	0.44(0.29–0.66)
Respiratory	0.68(0.58–0.80)	0.73(0.62–0.86)	0.43(0.32–0.58)	0.47(0.34–0.64)	0.72(0.60–0.85)	0.76(0.64–0.91)
Digestive	0.88(0.78–1.00)	0.97(0.85–1.11)	0.67(0.54–0.84)	0.96(0.76–1.20)	0.86(0.75–0.99)	0.89(0.77–1.03)
Genitourinary	0.40(0.32–0.52)	0.49(0.38–0.63)	0.23(0.14–0.39)	0.41(0.25–0.70)	0.42(0.32–0.54)	0.46(0.35–0.60)
Dermatologic	0.83(0.65–1.06)	0.81(0.63–1.04)	0.41(0.25–0.70)	0.49(0.29–0.83)	0.90(0.70–1.17)	0.84(0.65–1.10)
Musculoskeletal	0.73(0.64–0.84)	0.71(0.61–0.82)	0.52(0.41–0.66)	0.52(0.40–0.68)	0.75(0.65–0.87)	0.72(0.62–0.84)
Other	1.34(1.20–1.51)	1.05(0.91–1.22)	2.02(1.71–2.39)	1.32(1.07–1.63)	1.15(1.02–1.31)	0.97(0.83–1.13)
Visit related to injury versus not	0.75(0.70–0.81)	1.19(1.07–1.33)	0.52(0.46–0.58)	1.60(1.36–1.89)	0.84(0.78–0.92)	1.08(0.96–1.21)

* NHAMCS, National Hospital Ambulatory Medical Care Survey.

Note: The adjusted odds ratio (OR) was from a logistic regression including all variables in the table.

*DUD*, drug use disorder; *SUD*, substance use disorder; *AUD*, alcohol use disorder; *CI*, confidence interval; OR, odds ratio *NH*, non-Hispanic; *H*, Hispanic; *CHIP*, Children’s Health Insurance Program; *C*, celsius.

Note: The adjusted odds ratio (OR) was from a logistic regression including all variables in the table.

*DUD*, drug use disorder; *SUD*, substance use disorder; *AUD*, alcohol use disorder; *CI*, confidence interval; *OR*, odds ratio; *DBP*, diastolic blood pressure; *SBP*, systolic blood pressure.

**Table 4:** Proportion of Emergency Severity Index, hospital admission, ICU admission, medical resources utilization, stratified by alcohol/drugs substance use disorder, *NHAMCS 2016–2017.

	All	DUD Only	AUD Only	SUD (DID or AUD)	No SUD
ESI score		**	**	**	
1 (Immediate)	189(1.0)	20(1.0)	10(1.1)	21(0.9)	168(1.0)
2 (Emergent)	2,621(13.1)	348(17.8)	199(21.7)	439(18.3)	2,182(12.4)
3 (Urgent)	10,134(50.8)	999(51.1)	495(54.0)	1,231(51.2)	8,903(50.7)
4 (Semi-urgent)	6,046(30.3)	513(26.2)	181(19.8)	619(25.8)	5,427(30.9)
5 (Non-urgent)	959(4.8)	75(3.8)	31(3.4)	92(3.8)	867(4.9)
Hospital admission	3,854(14.0)	443(16.6)**	281(22.2)**	589(17.9)**	3,265(13.4)
ICU	469(1.7)	56(2.1)	37(2.9)**	74(2.3)**	395(1.6)
Death in ED or hospital	2857(10.4)	289(10.8)	192(15.2)**	395(12.0)**	2,462(10.1)
Left before/after triage	774(2.8)	88(3.3)	53(4.2)**	113(3.4)*	661(2.7)
Blood test performed	15,082(54.6)	1,610(60.3)**	924(73.0)**	2,054(62.6)**	13,028(53.6)
Any imaging performed	14,496(52.5)	1,181(44.3)**	578(45.7)**	1,505(45.9)**	12,991(53.4)
Radiograph in ED	9,805(35.5)	843(31.6)**	380(30.0)**	1,057(32.2)**	8,748(36.0)
CT in ED	5,737(20.8)	481(18.0)*	310(24.5)**	650(19.8)	5,087(20.9)
Ultrasound in ED	1,653(6.0)	107(4.0)**	47(3.7)**	136(4.1)**	1,517(6.2)
MRI in ED	307(1.1)	21(0.8)	6(0.5)*	25(0.8)*	282(1.2)
Other Imaging in ED	359(1.3)	30(1.1)	11(0.9)	36(1.1)	323(1.3)
Procedure	13,448(48.7)	1,254(47.0)*	591(46.7)	1,561(47.6)	11,887(48.9)

* NHAMCS, National Hospital Ambulatory Medical Care Survey.

Note: The missing proportion for waiting time is 15%, for ESI score is 28%. “Waiting time” refers to the time from arrival to seeing the physician

Independent test was performed on categories of drug use disorder (DUD), alcohol use disorder (AUD), and drug or alcohol use disorder. (SUD). Pearson’s Chi-squared test was performed on unweighted samples, and Rao-Scott corrected Chi-squared test was performed on weighted samples.

* P < 0.05,

** P < 0.01

*DUD*, drug use disorder; *SUD*, substance use disorder; *AUD*, alcohol use disorder; *ESI*, Emergency Severity Index; *ICU*, intensive care unit; *ED*, emergency department; *CT*, computed tomography; *MRI*, magnetic resonance imaging.

**Table 5:** Odds ratio of Emergency Severity Index, hospital admission, ICU admission, medical resources utilization for patients with vs without substance use disorder, *NHAMCS.

	SUD (AUD or DUD)		AUD Only		DUD Only	
			
	Crude OR (95% CI)	Adjusted OR (95% CI)	Crude OR (95% CI)	Adjusted OR (95% CI)	Crude OR (95% CI)	Adjusted OR (95% CI)
ESI Score: Immediate or Emergency vs semi to Non-urgent	1.73(1.53–1.97)	1.32(1.14–1.53)	2.58(2.12–3.13)	1.35(1.08–1.68)	1.65(1.43–1.89)	1.40(1.20–1.64)
ESI Score: Urgent vs. semi- or non-urgent	1.19(1.09–1.30)	1.19(1.08–1.31)	1.60(1.37–1.86)	1.40(1.19–1.66)	1.16(1.05–1.28)	1.20(1.08–1.33)
Hospital Admission	1.41(1.28–1.55)	1.28(1.14–1.43)	1.82(1.59–2.09)	1.23(1.05–1.44)	1.26(1.13–1.40)	1.22(1.08–1.38)
ICU	1.40(1.09–1.80)	1.40(1.05–1.85)	1.81(1.29–2.54)	1.18(0.81–1.73)	1.27(0.96–1.69)	1.46(1.07–1.99)
Death	1.22(1.09–1.36)	1.31(1.15–1.49)	1.59(1.36–1.86)	1.32(1.10–1.58)	1.06(0.93–1.20)	1.22(1.06–1.41)
Left before triage	1.28(1.04–1.56)	1.03(0.83–1.28)	1.55(1.17–2.07)	1.34(0.99–1.83)	1.21(0.96–1.51)	0.95(0.75–1.20)
Blood test	1.45(1.35–1.56)	1.58(1.44–1.73)	2.33(2.06–2.65)	2.46(2.13–2.86)	1.30(1.19–1.41)	1.40(1.27–1.54)
Any imaging	0.74(0.69–0.80)	0.84(0.77–0.91)	0.75(0.67–0.84)	0.80(0.71–0.91)	0.69(0.64–0.75)	0.81(0.74–0.89)
Radiograph	0.85(0.78–0.91)	0.89(0.82–0.97)	0.77(0.68–0.87)	0.70(0.61–0.80)	0.82(0.76–0.90)	0.91(0.82–1.00)
CT	0.93(0.85–1.02)	0.98(0.89–1.09)	1.25(1.10–1.43)	1.17(1.01–1.35)	0.82(0.74–0.91)	0.90(0.81–1.01)
Ultrasound	0.65(0.54–0.78)	0.89(0.73–1.07)	0.59(0.44–0.80)	1.10(0.80–1.50)	0.63(0.52–0.77)	0.82(0.67–1.02)
MRI	0.65(0.43–0.99)	0.79(0.51–1.21)	0.41(0.18–0.93)	0.43(0.19–0.98)	0.68(0.44–1.07)	0.85(0.53–1.35)
Procedure	0.93(0.86–1.00)	0.96(0.89–1.04)	0.91(0.82–1.02)	0.95(0.85–1.07)	0.90(0.84–0.98)	0.93(0.86–1.01)

* NHAMCS, National Hospital Ambulatory Medical Care Survey.

Note: “Demographic” includes gender, age group, and race/ethnicity; “socioeconomic” includes residence type, insurance type, and census region; “visiting & clinical” includes year, day of the week, arrival by ambulance, seen within last 72 hours, pain level, temperature, heart rate, diastolic blood pressure, injury status, and reason for visit.

*SUD*, substance use disorder; *AUD*, alcohol use disorder; *DUD*, drug use disorder; *OR*, odds ratio; *ESI*, Emergency Severity Index; *ICU*, intensive care unit; *ED*, emergency department; *CT*, computed tomography; *MRI*, magnetic resonance imaging.
