# Supplementary material for: Emergency Department Visits by Patients with Substance Use Disorder in the United States
Source: West J Emerg Med. 2021 Aug 19;22(5):1076–85. doi: 10.5811/westjem.2021.3.50839 (PMC8463055; doi:10.5811/westjem.2021.3.50839)
Supplement: Supplementary file 1 [file wjem-22-1076-s001.docx]

Supplement Table 1. Baseline characteristics of patients presenting to the emergency department, stratified by alcohol/ drugs substance use disorder, *NHAMCS 2016–2017 (weighted sample).

|  | All | DUD Only | AUD Only | SUD (DUD or AUD) | No SUD |
| --- | --- | --- | --- | --- | --- |
|  | 215,240,594 | 19,191,999(8.9) | 8,779,847(4.1) | 23,881,116(11.1) | 191,360,000(88.9) |
| Male | 92,150,415(42.8) | 10,319,931(53.8)** | 5,904,335(67.2)** | 13,362,397(56.0)** | 78,788,018(41.2) |
| Age |  | ** | ** | ** |  |
| 18-25 | 31,677,848(14.7) | 3,082,996(16.1) | 826,424(9.4) | 3,461,502(14.5) | 28,216,346(14.7) |
| 26-39 | 58,487,071(27.2) | 6,471,848(33.7) | 1,983,684(22.6) | 7,544,252(31.6) | 50,942,819(26.6) |
| 40–49 | 32,430,413(15.1) | 3,499,457(18.2) | 1,521,894(17.3) | 4,300,947(18.0) | 28,129,466(14.7) |
| 50–59 | 33,595,252(15.6) | 3,622,299(18.9) | 2,586,250(29.5) | 4,888,029(20.5) | 28,707,223(15.0) |
| 60–74 | 34,864,200(16.2) | 2,089,758(10.9) | 1,686,590(19.2) | 3,101,267(13.0) | 31,762,933(16.6) |
| ≥ 75 | 24,185,810(11.2) | 425,640(2.2) | 175,005(2.0) | 585,118(2.5) | 23,600,692(12.3) |
| Race/ethnicity |  | ** |  | ** |  |
| NH White | 103,482,638(61.0) | 9,517,655(66.4) | 4,461,047(65.2) | 11,976,994(66.6) | 91,505,644(60.4) |
| H White | 13,708,707(8.1) | 854,465(6.0) | 454,330(6.6) | 1,098,023(6.1) | 12,610,684(8.3) |
| NH Black | 39,425,837(23.3) | 3,104,395(21.7) | 1,416,340(20.7) | 3,880,560(21.6) | 35,545,277(23.4) |
| H Black | 535,701(0.3) | 46,117(0.3) | 38,079(0.6) | 71,206(0.4) | 464,495(0.3) |
| Hispanic (other) | 7,622,180(4.5) | 409,519(2.9) | 312,963(4.6) | 530,653(2.9) | 7,091,527(4.7) |
| Asian | 3,114,715(1.8) | 167,012(1.2) | 59,634(0.9) | 188,225(1.0) | 2,926,490(1.9) |
| Other | 1,687,547(1.0) | 231,909(1.6) | 102,817(1.5) | 248,143(1.4) | 1,439,404(0.9) |
| Residence type |  | ** | ** | ** |  |
| Private residence | 199,456,518(95.0) | 16,976,022(91.2) | 7,304,999(86.1) | 20,974,159(90.4) | 178,482,359(95.5) |
| Nursing home | 4,326,842(2.1) | 68,231(0.4) | 100,018(1.2) | 153,161(0.7) | 4,173,681(2.2) |
| Homeless | 2,310,912(1.1) | 1,102,382(5.9) | 706,635(8.3) | 1,336,615(5.8) | 974,297(0.5) |
| Other | 3,907,893(1.9) | 459,756(2.5) | 369,337(4.4) | 728,041(3.1) | 3,179,851(1.7) |
|  |  | ** | ** | ** |  |
| Private insurance | 54,212,897(28.1) | 3,858,073(22.8) | 1,734,853(22.6) | 4,847,050(23.0) | 49,365,847(28.8) |
| Medicare | 51,235,274(26.6) | 2,678,708(15.8) | 1,659,905(21.6) | 3,814,756(18.1) | 47,420,518(27.6) |
| Medicaid or CHIP | 58,963,047(30.6) | 7,729,785(45.6) | 3,142,773(40.9) | 9,278,472(44.0) | 49,684,575(28.9) |
| Uninsured | 20,699,501(10.7) | 2,048,168(12.1) | 870,656(11.3) | 2,343,674(11.1) | 18,355,828(10.7) |
| Other | 7,636,327(4.0) | 635,665(3.8) | 281,781(3.7) | 788,559(3.7) | 6,847,768(4.0) |
| Day of ED Visit | ** | ** | ** |  |  |
| Weekend | 56,769,038 (26.4) | 5,484,027(28.6) | 2,460,934 (28.0) | 6,775,748 (28.4) | 49,993,290 (26.1) |
| Weekdays | 158,471,555 (73.6) | 13,707,972 (71.4) | 6,318,912 (72.0) | 17,105,368 (71.6) | 141,366,187 (73.9) |
| Arrive by ambulance | 39,310,535(18.7) | 4,968,329(26.3)** | 4,040,270(47.1)** | 7,109,665(30.3)** | 32,200,869(17.3) |
| Seen within last 72 hours | 6,121,292(3.1) | 734,332(4.2)* | 407,479(5.1)** | 958,972(4.4)** | 5,162,320(2.9) |
| Pain level at Presentation |  | ** | ** | ** |  |
| No pain | 34,913,498(23.2) | 3,257,286(25.8) | 2,057,638(37.4) | 4,322,315(27.5) | 30,591,183(22.7) |
| Mild | 14,203,227(9.4) | 930,504(7.4) | 488,680(8.9) | 1,277,407(8.1) | 12,925,820(9.6) |
| Moderate | 46,610,296(30.9) | 3,473,993(27.5) | 1,284,042(23.3) | 4,330,471(27.5) | 42,279,825(31.3) |
| Severe | 54,927,003(36.5) | 4,968,751(39.3) | 1,677,675(30.5) | 5,789,146(36.8) | 49,137,858(36.4) |
| Temperature at Presentation | 36.8(38.0) | 36.7(34.7) | 36.7(35.7) | 36.8(35.5) | 36.8(38.3) |
| Heart Rate at Presentation | 86.1(1557.0) | 89.5(1515.0) | 92.4(1559.6) | 90.1(1537.2) | 85.6(1553.9) |
| DBP at Presentation | 80.5(1292.6) | 82.8(1218.0) | 83.3(1298.3) | 83.0(1259.6) | 80.2(1294.4) |
| SBP at Presentation | 138.0(2115.5) | 136.1(1820.1) | 135.1(1867.0) | 136.1(1868.8) | 138.3(2145.6) |
| Census Region |  |  |  |  |  |
| Northeast | 33,427,214(15.5) | 2,279,091(11.9) | 1,625,223(18.5) | 2,967,565(12.4) | 30,459,649(15.9) |
| Midwest | 52,153,223(24.2) | 6,214,715(32.4) | 2,194,080(25.0) | 7,534,396(31.5) | 44,618,828(23.3) |
| South | 83,789,428(38.9) | 6,902,935(36.0) | 2,578,176(29.4) | 8,229,621(34.5) | 75,559,807(39.5) |
| West | 45,870,728(21.3) | 3,795,257(19.8) | 2,382,367(27.1) | 5,149,534(21.6) | 40,721,194(21.3) |
| Visit Related to Injury | 65,644,383(30.5) | 6,360,692(33.1) | 4,012,313(45.7) | 8,449,042(35.4) | 57,195,341(29.9) |
| Reason for ED Visit |  | ** | ** | ** |  |
| General | 41,926,806(19.5) | 3,868,597(20.2) | 1,751,058(20.0) | 4,721,532(19.8) | 37,205,274(19.5) |
| Psychiatric | 7,841,631(3.7) | 2,123,253(11.1) | 1,502,220(17.1) | 2,813,368(11.8) | 5,028,263(2.6) |
| Neurologic | 15,867,786(7.4) | 1,291,678(6.7) | 503,334(5.7) | 1,519,347(6.4) | 14,348,438(7.5) |
| Cardiovascular and lymphatic | 4,601,441(2.1) | 428,573(2.2) | 165,638(1.9) | 492,990(2.1) | 4,108,451(2.2) |
| Eyes and/or ears | 4,397,893(2.1) | 223,305(1.2) | 22,625(0.3) | 245,930(1.0) | 4,151,963(2.2) |
| Respiratory | 21,990,645(10.2) | 1,517,431(7.9) | 432,867(4.9) | 1,862,171(7.8) | 20,128,474(10.5) |
| Digestive | 35,521,482(16.5) | 2,923,833(15.2) | 1,214,954(13.8) | 3,785,363(15.9) | 31,736,119(16.6) |
| Genitourinary | 10,469,363(4.9) | 462,086(2.4) | 80,099(0.9) | 518,525(2.2) | 9,950,838(5.2) |
| Dermatologic | 6,390,594(3.0) | 557,101(2.9) | 66,209(0.8) | 601,327(2.5) | 5,789,267(3.0) |
| Musculoskeletal | 32,172,694(15.0) | 2,250,644(11.7) | 573,757(6.5) | 2,566,838(10.8) | 29,605,856(15.5) |
| Other | 33,769,883(15.7) | 3,528,464(18.4) | 2,462,801(28.1) | 4,736,691(19.8) | 29,033,191(15.2) |

*NHAMCS, National Hospital Ambulatory Medical Care Survey.

Independent test was performed on categories of drug use disorder (DUD), alcohol use disorder (AUD), and drug or alcohol use disorder (SUD). Pearson's chi-squared test was performed on unweighted samples, and Rao-Scott corrected chi-squared test was performed on weighted samples. **P* < 0.05, ***P* < 0.01.

*SUD,* substance use disorder*; AUD,* alcohol use disorder*; DUD,* drug use disorder*; NH*, non-Hispanic; *H*, Hispanic; *CHIP*, Children’s Health Insurance Program.

Supplement Table 2. Proportion of Emergency Severity Index, hospital admission, ICU admission, medical resources utilization, stratified by alcohol/ drugs SUD , NHAMCS 2016-2017 (weighted sample).

|  | All | DUD Only | AUD Only | SUD (DUD or AUD) | No SUD |
| --- | --- | --- | --- | --- | --- |
| ESI score |  | ** | ** | ** |  |
| 1 (Immediate) | 1,474,896(1.0) | 149,392(1.1) | 68,572(1.1) | 158,033(0.9) | 1,316,863(1.0) |
| 2 (Emergent) | 21,345,538(14.0) | 2,707,641(19.7) | 1,512,538(24.7) | 3,452,525(20.4) | 17,893,013(13.2) |
| 3 (Urgent) | 76,939,174(50.3) | 6,827,511(49.8) | 3,155,661(51.4) | 8,422,863(49.7) | 68,516,310(50.4) |
| 4 (Semi-urgent) | 46,377,544(30.3) | 3,572,159(26.0) | 1,213,773(19.8) | 4,326,381(25.5) | 42,051,162(30.9) |
| 5 (Non-urgent) | 6,877,151(4.5) | 458,338(3.3) | 185,250(3.0) | 586,214(3.5) | 6,290,937(4.6) |
| Hospital Admission | 30,582,913(14.2) | 3,262,137(17.0)** | 2,130,599(24.3)** | 4,439,149(18.6)** | 26,143,764(13.7) |
| ICU | 3,852,739(1.8) | 412,099(2.1)** | 272,256(3.1) | 525,336(2.2) | 3,327,403(1.7) |
| Death in ED or hospital | 23,067,834(10.7) | 2,207,839(11.5) | 1,586,580(18.1)** | 3,149,783(13.2)** | 19,918,051(10.4) |
| Left before/after triage | 6,245,255(2.9) | 646,272(3.4) | 366,872(4.2) | 886,830(3.7) | 5,358,426(2.8) |
| Blood test performed | 119,303,863(55.4) | 11,878,081(61.9)** | 6,498,779(74.0)** | 15,211,844(63.7)** | 104,092,019(54.4) |
| Any imaging performed | 117,181,932(54.4) | 9,419,177(49.1)** | 4,314,333(49.1)** | 11,933,746(50.0)** | 105,248,187(55.0) |
| Radiograph in ED | 80,087,700(37.2) | 6,589,295(34.3)* | 2,784,548(31.7)** | 8,321,441(34.8)* | 71,766,259(37.5) |
| CT in ED | 46,480,608(21.6) | 3,828,974(20.0) | 2,295,637(26.1)** | 5,066,083(21.2) | 41,414,525(21.6) |
| Ultrasound in ED | 12,996,039(6.0) | 938,436(4.9) | 376,374(4.3) | 1,192,463(5.0) | 11,803,576(6.2) |
| MRI in ED | 2,607,475(1.2) | 178,809(0.9) | 53,440(0.6) | 211,362(0.9) | 2,396,113(1.3) |
| Other Imaging in ED | 2,394,747(1.1) | 196,642(1.0) | 54,273(0.6) | 235,114(1.0) | 2,159,632(1.1) |
| Procedure | 106,962,016(49.7) | 9,490,036(49.4) | 4,377,022(49.9) | 11,927,492(49.9) | 95,034,524(49.7) |

Independent test was performed on categories of drug use disorder (DUD), alcohol use disorder (AUD), and drug or alcohol use disorder (SUD). Pearson's chi-squared test was performed on unweighted samples, and Rao-Scott corrected chi-squared test was performed on weighted samples. **P* < 0.05, ***P* < 0.01.

*SUD,* substance use disorder*; AUD,* alcohol use disorder*; DUD,* drug use disorder*; ESI,* Emergency Severity Index*; ICU,* intensive care unit*; ED,* emergency department*; CT,* computed tomography*; MRI,* magnetic resonance imaging*.*
